# The effect of familiarity on infants’ social categorization capacity

**DOI:** 10.1371/journal.pone.0247710

**Published:** 2021-03-04

**Authors:** Matar Ferera, Anthea Pun, Andrew Scott Baron, Gil Diesendruck

**Affiliations:** 1 Department of Psychology, Bar-Ilan University, Ramat-Gan, Israel; 2 Gonda Brain Research Center, Bar-Ilan University, Ramat-Gan, Israel; 3 Department of Psychology, University of British Columbia, Vancouver, Canada; Ghent University, BELGIUM

## Abstract

Recent studies indicate that a preference for people from one’s own race emerges early in development. Arguably, one potential process contributing to such a bias has to do with the increased discriminability of own- vs. other-race faces–a process commonly attributed to perceptual narrowing of unfamiliar groups’ faces, and analogous to the conceptual homogenization of out-groups. The present studies addressed two implications of perceptual narrowing of other-race faces for infants’ social categorization capacity. In Experiment 1, White 11-month-olds’ (*N* = 81) looking time at a Black vs. White face was measured under three between-subjects conditions: a baseline “preference” (i.e., without familiarization), after familiarization to Black faces, or after familiarization to White faces. Compared to infants’ a priori looking preferences as revealed in the baseline condition, only when familiarized to Black faces did infants look longer at the "not-familiarized-category" face at test. According to the standard categorization paradigm used, such longer looking time at the novel (i.e., "not-familiarized-category") exemplar at test, indicated that categorization of the familiarized faces had ensued. This is consistent with the idea that prior to their first birthday, infants already tend to represent own-race faces as individuals and other-race faces as a category. If this is the case, then infants might also be less likely to form subordinate categories within other-race than own-race categories. In Experiment 2, infants (*N* = 34) distinguished between an arbitrary (shirt-color) based sub-categories only when shirt-wearers were White, but not when they were Black. These findings confirm that perceptual narrowing of other-race faces blurs distinctions among members of unfamiliar categories. Consequently, infants: a) readily categorize other-race faces as being of the same kind, and b) find it hard to distinguish between their sub-categories.

## Introduction

Social categorization is a ubiquitous phenomenon. People across cultures divide their social world into groups based on a variety of criteria–gender, race, religion, ethnicity, etc.–and biases, prejudice, and even conflicts often arise based on such groupings. Recent studies reveal that such biases may be manifest even in prelinguistic infants. For instance, by 1-year of age, infants more easily associate positively valenced stimuli with individuals who speak their own language, than with individuals who speak another language [1], and associate positively valenced music with own-race faces and negatively valenced music with other-race faces [2]. In other words, by 1 year of age, infants seem capable of representing certain social groups, and have valenced attitudes towards them [see also 3, 4].

Developmental studies indicate that social categorization skills are necessary precursors to intergroup biases [e.g., 5–7], though as social psychologists note, such skills might not be sufficient [see 8, 9]. That is, for intergroup biases to arise, perceivers need to categorize people into groups, but categorization alone may not lead to bias (e.g., one may have categories of Danes and of Swedes, but not prefer one over the other). More strongly associated with such biases are asymmetric construals of social categories. In particular, one process that arguably underlies adults’ inter-group biases is the so-called out-group homogeneity effect [10–12]; namely, the tendency to conceive of members of one’s in-group as diverse individuals, and members of one’s out-group as indistinguishable exemplars of the category [13, 14]. The argument is that conceiving of out-groups as homogeneous, facilitates stereotyping and generalization of characteristics to an entire group. Recent studies indicate that the out-group homogeneity effect is present in 5-year-old children [15], and that an intervention aimed at perceptually individuating other-race members–arguably an opposite process to categorization–reduced implicit racial bias in preschool children [16, 17]. Given the appearance of inter-group biases already in infancy, the goal of the present studies was to investigate whether an asymmetry akin to the out-group homogeneity effect is present in 1-year-olds. There is already some extant evidence consistent with this possibility, coming from two related lines of research. First, a number of studies have documented the development of the other-race effect in infants. In particular, studies have shown that robustly by 9-months of age, infants become better capable of recognizing individuals from their own-race than individuals from unfamiliar races [18–20]. Arguably, due to scant exposure to exemplars from unfamiliar racial groups, infants undergo a process of perceptual narrowing, becoming less adept at processing individuating features of faces of those unfamiliar groups–while maintaining their capacity to process such features in faces of their familiar group. In fact, infants’ visual scanning patterns vary as a function of the racial familiarity of the faces [21–23], pointing to a possible mechanism underlying an advantage with individuating familiar (compared to unfamiliar) kinds of faces.

The implication to categorization deriving from the above phenomenon is that, at least by 9-months of age, other-race faces are seen as more similar to each other than own-race faces. Consequently, infants might readily lump other-race faces into a group, thus forming a category [see e.g., 24]. A recent study showed that both adults and children evinced this so-called other-race *categorization* advantage [25]. And indeed, a second line of evidence consistent with this hypothesis derives from the handful of studies assessing infants’ social categorization capacities [e.g., 26, 27on gender; 28on age; and 29, 30on race]. Especially pertinent to the present hypothesis, studies found that infants tend to show an asymmetry in categorizing familiar vs. unfamiliar categories [see 27on gender, 28on age]. Particularly in regard to race, 6-month-old Caucasian infants looked longer at a Caucasian face after being familiarized to Asian faces, but did not look longer at an Asian face after being familiarized to Caucasian faces [29, see also 30]. These findings suggest that infants’ capacity to form a social category depends on the nature of the stimuli, and specifically, on the extent of familiarity with the stimuli. It seems that in the case of race, infants readily perceive unfamiliar-race faces as constituting a category.

In the present two studies we addressed this stipulation about unfamiliar-race faces directly. In particular, we first asked whether infants are more likely to perceive a set of unfamiliar–as opposed to familiar–group’s exemplars as a category (Experiment 1). We then addressed whether given the presumed construal of unfamiliar groups as *a* homogeneous group, infants may be less likely to differentiate *sub*-groups within an unfamiliar group than within a familiar one (Experiment 2).

Crucially, in designing Experiment 1, we considered a few important caveats raised in the literature. In particular, reviewing the findings on infants’ social categorization, Quinn, Lee, & Pascalis [31] recently noted that there seem to be robust conclusions to be drawn regarding the development of categorization of other-races (e.g., Caucasian infants’ categorization of Asian vs. Black faces, as in Quinn et al. [30]). In contrast, they state, “*with regard to a category of own-race faces, the evidence remains fragile. As was the case in the investigation conducted by Anzures and colleagues [29], the novel category preference for other-race faces after familiarization with own-race faces* [here referring to the findings from Quinn et al. [30]] *could have arisen because of an a priori preference*” (p. 174). To clarify, for instance, Anzures et al.’s [29] finding that infants looked longer at a Caucasian face after being familiarized to Asian faces could have resulted either because infants perceived the Asian familiarization faces as more alike and thus more easily grouped them into a category, or because the Caucasian face shown at test was more attractive to the infants *irrespective* of what they saw during familiarization. Corroborating this interpretive puzzle, there *are* in fact differences in a priori preferences to faces from familiar vs. novel racial groups, which change throughout the first year of life [22, 32, 33]. Quinn et al. [31] make the point primarily in regard to own-race faces, but the more general point is that the design applied thus far for investigating infants’ racial categorization capacity, does not allow decisive conclusions regarding said capacity.

In order to uncover conclusively whether this asymmetry derives from differential categorization capacities of familiar vs. unfamiliar races, it is imperative to first determine infants’ baseline looking preferences towards faces from the different races [34]. Then, one can assess whether different familiarization sequences significantly alter these baseline preferences. Only if they do, one can infer that categorization has ensued. Indeed, this was the strategy adopted by Damon et al. [28] in their study on infants’ age categorization, and Quinn et al. [27] in their study on infants’ gender categorization. The former found no baseline preferences, from which they concluded that infants’ looking preferences after familiarization must have reflected categorization. The latter did find a baseline preference for looking at female faces, from which they determined that the looking preferences manifested at test could not be taken as conclusive evidence for categorization. In fact, to that end, we believe that once baseline preferences are assessed, a second crucial step to more decisively assess infants’ categorization capacity is to *directly compare* potential changes relative to that baseline, in infants’ looking preferences after familiarization. And as Oakes [34] further recommended, it is best to present two groups of infants–one undergoing familiarization and another not–with the exact same stimuli. The present Experiment 1 does that, in the context of racial categorization.

Experiment 1 was modelled after Quinn et al. [30]. Namely, we familiarized White 11-month-old infants to a series of faces from a particular race (e.g., White), and then at test presented two new faces, one from the same category as the familiarization exemplars (White), and the other from the contrasting category (Black). Our main measure of interest was the proportion of looking time at either face. In order to assess the effect of familiarity, a set of infants was familiarized to faces from a familiar race (i.e., White adults) and another set to faces from an unfamiliar race (i.e., Black adults). Importantly, expanding upon the work of Quinn et al., we followed Oakes’ [34] recommendation, and added a Baseline condition to the design, one in which a set of infants did not undergo any familiarization, and were simply exposed to the same pair of pictures presented during the test phase of the other conditions. We then directly compared infants’ looking patterns across these three conditions. Following Oakes’s [34] analysis, this design allowed us to assess how familiarization affected, if at all, infants’ a priori preferences for looking at a White vs. a Black face.

The assumption was that upon seeing a series of faces from a familiar race (White adults), White infants would be quite capable of perceiving them as distinct individuals. Consequently, they would be less likely to focus on the similarities among them, and thus less likely to construe them as a category. Further, we reasoned that infants’ looking pattern after familiarization would not be significantly different from the pattern revealed by infants in the baseline condition. In contrast, upon seeing a series of faces from an unfamiliar race (Black adults), infants would be less capable of perceiving them as distinct individuals. Consequently, they would be more likely to perceive the similarities among them, and thus construe them as a category. In that case, their looking pattern would be significantly different from that manifested by infants in the baseline condition. In particular, the hypothesis was that by forming a category of Black adults, infants in this condition would look longer at an exemplar from a contrasting category compared to infants in the baseline condition [see e.g., 35, 36].

## Experiment 1: Racial categorization

Experiment 1 was designed to assess whether there are differences in the capacity of infants to represent a social category based on familiar vs. unfamiliar groups’ exemplars. To that end, we decided to focus on race-based categories, as we could be certain that for our participants–Israeli White infants–White exemplars would be substantially more familiar than Black exemplars. The design included three between-subjects conditions. Infants in the Baseline condition simply saw a pair of faces presented simultaneously: a Black adult next to a White adult. We measured the proportion of time infants looked at the two faces. In the two familiarization conditions, infants first saw 9 exemplars of either White or Black adults, and then saw the same pair of faces infants in the Baseline condition saw. Again we measured the proportion of time infants looked at the two faces. The typical index of categorization in such a paradigm is whether after familiarization infants look longer at the contrasting category (e.g., whether after being familiarized to Black faces, infants then look longer at the White test face than the Black test face). However, in order to correct for infants’ a priori preference, the index of categorization needs to compare not the proportions of looking at one face vs. another at test, but rather the pattern of looking at the two test faces to the pattern revealed at baseline. Based on previous studies [22, 33], we expected that the a priori preference of infants at this age, was to look longer at the unfamiliar (i.e., Black) face. Thus the hypothesis was that if infants more readily construe faces from an unfamiliar race as a category, then infants’ looking time pattern at test should differ the most–compared to the baseline–after exposure to Black than to White familiarization exemplars. In other words, whereas infants’ preference for looking at a Black face after exposure to White faces during familiarization would be the same as their baseline preference, this preference would be substantially lower after exposure to Black faces during familiarization.

Experiment 1 also manipulated two additional factors that had previously been shown to impact infants’ categorization capacity. One general factor is the presence or absence of labeling. Previous studies showed that giving a common label to various exemplars facilitated infants’ formation of object and animal categories [35–37], and toddlers’ formation of various social categories [38]. We followed Balaban and Waxman’s protocol, and for part of the infants provided a label during the presentation of the familiarization exemplars, and for the other part did not. The hypothesis was that infants in the Label condition would succeed in detecting racial categories more than infants in the No-label condition (i.e., across all other factors). The other factor had to do with the gender of the exemplars being categorized. Previous studies have shown that infants’ preference and discriminability of own- vs. other-race faces is especially prominent when targets are women [39, 40]. Thus here, a group of infants was familiarized with Black or White men, and the others with Black or White women. Thus, if indeed targets’ gender contributes to racial categorization, we would expect infants to evince categorization of women more than of men (i.e., across all other factors).

### Method

All experiments were approved by the Ethics Committee of the Department of Psychology of Bar-Ilan University. Although the experiments were not pre-registered, sample sizes were determined based on previous studies (see below), all the variables collected are reported here, and choice of measures and analyses were predetermined, based on standard procedures in the field, and following the rationale expounded in the Procedures of both experiments.

### Participants

Eighty-one Israeli White 11-month-old infants (34 girls; mean age = 10.9 months; range = 8.9 months to 13 months) constituted the final sample of participants. Twelve additional infants were excluded due to fussiness during the experiment (6) or experimenters’ error (6). Fussiness behaviors included crying and restlessness that resulted in inability to watch the presentation or inability to code children’s looking behavior. Experimenters’ error included wrong operation of the experimental setup (e.g., leaving the mouse arrow on the screen). Sample size was planned in advance, determined according to previous studies in this area [28, 30, 36]. These studies reported effect sizes of approximately *Cohen’s f* = 0.4 (conventionally considered a large effect), and thus using this as an estimate, and setting α = 0.05, our sample sizes rendered an estimated power of approximately 0.8. These power estimates refer only to tests of main effects. Families were all White and recruited from the greater Ramat-Gan area, which is predominantly White in terms of racial composition. Families were recruited via ads in and around the university, and via Facebook. As compensation for their participation, families received a baby-shirt with the lab’s logo and a voucher for a bookstore.

### Design

Infants were assigned to one of three experimental conditions: Baseline (*n* = 26), White familiarization (*n* = 29), and Black familiarization (*n* = 26). Within these three conditions, we also varied the gender of the targets: Baseline (*n*_*women*_ = 13, *n*_*men*_ = 13), White familiarization (*n*_*women*_ = 15, *n*_*men*_ = 14), Black familiarization (*n*_*women*_ = 17, *n*_*men*_ = 9). Within the White and Black familiarization conditions we also varied the presence of labelling: White familiarization (*n*_*Label*_ = 15, *n*_*No*-*label*_ = 14), Black familiarization (*n*_*Label*_ = 16, *n*_*No-label*_ = 10).

### Materials

The stimuli were pictures of Black and White adult faces with a neutral expression. Pictures were 15–18 cm wide and 22cm high, presented on a grey background. Most pictures were taken from the NimStim database. Stimuli were validated by adults’ ratings on scales of attractiveness and variance. Specifically, 94 White adults rated the pictures (using an online Qualtrics survey): Half (25 women and 23 men, *M*_*age*_ = 34, *age range* = 22–70) rated the White stimuli, and half (27 women and 19 men, *M*_*age*_ = 36, *age range* = 25–75) the Black stimuli. All raters were first presented with the attractiveness question and then with the variance question. For the attractiveness validation, raters were presented with ten faces, one at a time, and were asked: "How attractive is the person in this picture?" Answers were given on a scale from 1 (not at all) to 7 (extremely attractive). We found no difference in attractiveness ratings between the White (*M* = 4.63) and Black (*M* = 4.52) faces, *t*(94) = -0.494, *p* = 0.622. For the variance validation, raters were presented with ten pairs of faces, namely the nine familiarization and one test slides that appeared consecutively in the experiments’ presentation. Raters were asked: "How similar are these two pictures to one another?" Answers were given on a scale from 1 (not at all) to 7 (extremely similar). We found no difference in the similarity ratings between the White (*M* = 5.18) and Black (*M* = 5.20) faces, *t*(94) = 0.146, *p* = 0.88.

### Procedure

Parents arrived at the laboratory, where they were informed about the study’s procedure and goal, and were asked to sign a consent form permitting their infants to participate in the study. After a short warm-up session in a play room, infants and parents moved to a testing room, and infants were sat on their parent’s lap in front of a 42” screen. A webcam located below the screen recorded infants’ looking time (for later coding). Parents were instructed not to interfere and to close their eyes during the test trial.

**Fig 1:**
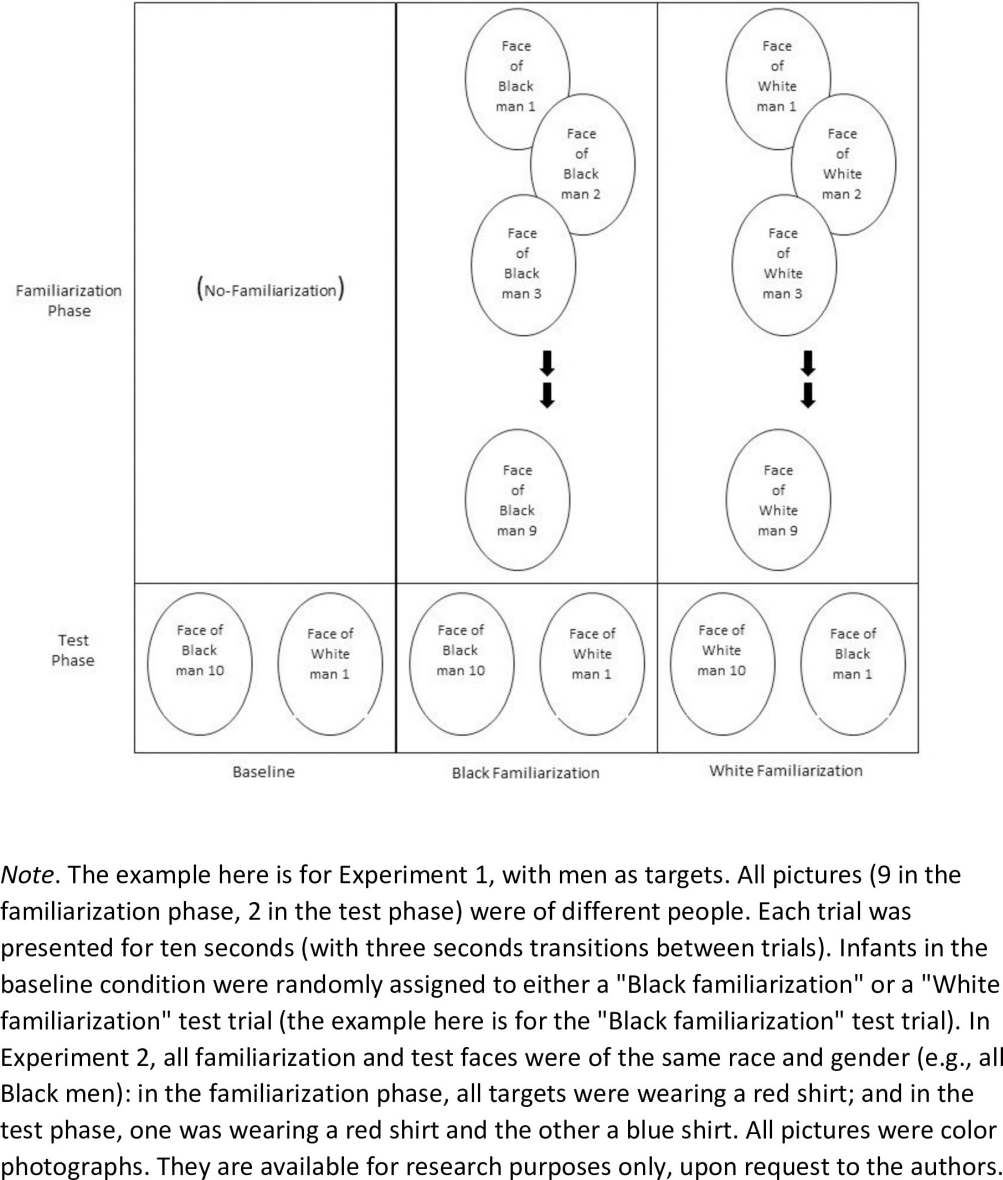
Schematic display of the procedure used in the categorization task in Experiment 1.

Infants in the baseline condition were presented with a single test trial, identical to the test trial used in the familiarization conditions. Infants in the two familiarization conditions started with a familiarization phase, in which they saw a series of nine photos one at a time depicting adults–either men or women–of one of the two contrasting categories (i.e., nine different Black or White adults). Following Balaban & Waxman’s [35] procedure, for infants in the Label condition, six of the familiarization exemplars were labeled by the same made-up word (“Look, Zergon”); for infants in the No-label condition, trials were presented in silence. In each of the nine familiarization trials, the photo appeared at the center of the screen, for 10 seconds (with three seconds transitions between trials). After the nine familiarization trials, infants in both conditions saw a test trial, consisting of the same two faces presented in the Baseline condition (all infants saw the same test trial–either men or women, according to the infants’ target gender condition). The faces appeared simultaneously, side by side (with approximately 27cm space between them), for 10 seconds. The faces were of another exemplar from the familiarized category (e.g., another Black adult), and an exemplar from the contrasting category (e.g., a White adult; see Fig 1 for a schematic display of the procedure).

Given our goal to assess the effect of familiarization beyond infants’ potential a priori preference for familiar vs. unfamiliar-race faces, we used as our dependent measure the percentage of looking time at the Black test exemplar, out of the total looking time at both test exemplars (the percentage of looking at the White is the direct complement of that). Thus, if as hypothesized, infants would more readily construe a category based on other-race familiarization exemplars, then we would expect infants in the Black Familiarization condition to look at their contrasting test exemplar (i.e., the White adult) more than they had done in the Baseline condition. In other words, the percentage of looking at the Black exemplar would be lower in the Black Familiarization than in the Baseline condition.

Coding was performed offline by trained RAs, unaware of experimental conditions. For each trial, a coder decided frame by frame whether the infant’s gaze was directed to the left, center, or right part of the screen. A second coder independently coded a random 20% of subjects, achieving 96% agreement between coders.

### Results and discussion

#### Preliminary analysis

We conducted a preliminary analysis in order to assess whether infants in the Baseline condition had an a priori preference for looking at Black vs. White faces. A one sample t-test against a 50% criterion (i.e., chance value) showed that infants looked at the Black face significantly longer than expected by chance (*M* = 58%, *SE* = 2.0%, *N* = 26; *t*(25) = 4.041, *p*<0.001, *r* = 0.628). Thus, consistent with previous findings [22], after 9-months of age, infants are more attracted to faces of other-race adults.

#### Main analyses

**Fig 2:**
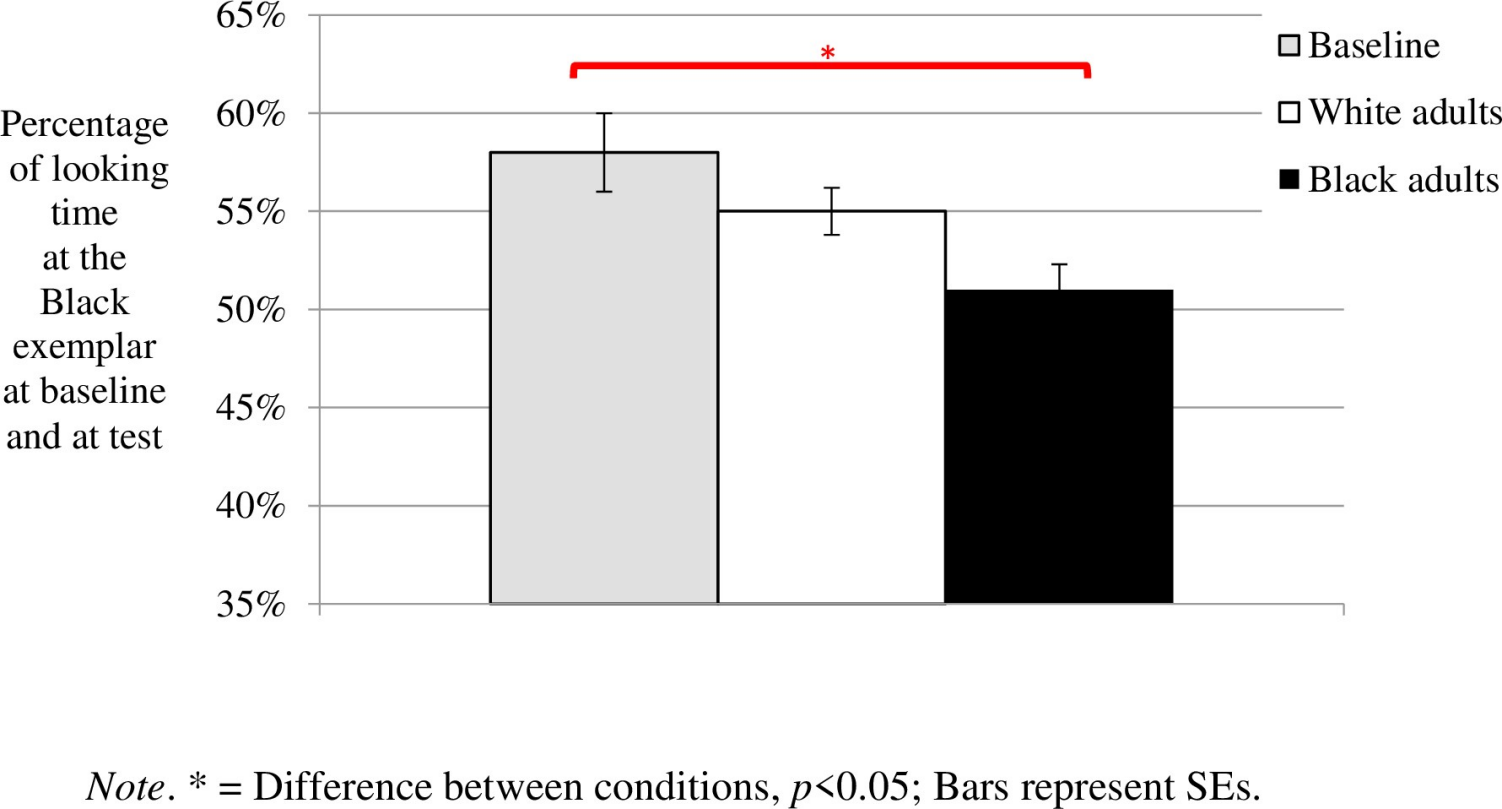
Results of Experiment 1 (Race).

Our main analysis was an ANOVA including the three familiarization stimuli conditions (Baseline, Black Familiarization, White Familiarization) and the two targets’ gender conditions (women, men) as independent factors, and the proportion of looking time at the Black face as the dependent measure. The ANOVA revealed only a significant main effect of familiarization stimuli conditions, *F*(2,75) = 4.248, *p* = 0.018, *η*^*2*^ = 0.102, *1-β* = 0.727, with Scheffe post-hoc tests indicating a significant difference between the Black Familiarization and the Baseline conditions (*p* = 0.042, *Cohen’s d* = 0.619), but not between the other conditions (between the White Familiarization and the Baseline conditions, *p* = 0.567, *Cohen’s d* = 0.336; between the White Familiarization and the Black Familiarization conditions, *p* = 0.295, *Cohen’s d* = 0.423; see Fig 2). In sum, compared to their baseline preference, infants looked significantly less at a Black test exemplar after being familiarized to Black exemplars, but not after being familiarized to White exemplars.

As a final analysis, we classified infants as to whether they looked at the Black test exemplar more or less than at the White test exemplar. Non-parametric tests comparing the number of infants of these two classes among conditions revealed a significant difference, *χ*^*2*^(2, *n* = 81) = 6.608, *p* = 0.037. In fact, whereas the proportion of infants who looked longer at the Black exemplar in the Baseline (77%) and White familiarization (79%) conditions were significantly different from chance distribution (*χ*^*2*^(1, *n* = 26) = 7.538, *p* = 0.006, and *χ*^*2*^(1, *n* = 29) = 9.966, *p* = 0.002, respectively), this was not the case in the Black familiarization condition (50%; *χ*^*2*^(1, *n* = 26) = 0.0, *p* = 1.0).

An additional analysis targeted the effect of labeling, which was relevant only for the two familiarization conditions. An ANOVA including labeling condition, familiarization condition, and targets’ gender condition revealed neither a significant main effect of labeling, nor any interaction involving labeling, all pertinent *F*s < 1.1, *p*s > 0.3.

Taken together, the main finding is that after being familiarized to nine faces of Black adults, and then shown a face of an additional White adult and a face of a Black adult, infants did *not* continue to show a preference for the Black adult. Familiarization to Black adults changed infants’ a priori looking preference, such that infants now increased their proportionate looking at the contrasting category (White) test exemplar. Infants recognized the Black familiarization faces as being of the same kind, and as different from the White test exemplar; they represented a category of Blacks, exclusive of Whites. In turn, after being familiarized to nine faces of White adults, and then shown a face of an additional White adult and a face of a Black adult, infants continued to show a preference for the Black adult similar to their a priori preference. Being familiarized to nine White adults did not make the Black adult at test any more salient than it was at Baseline. We will discuss this issue in the General Discussion.

The above conclusion confirms that perceptual narrowing of other-race faces not only sharpens infants’ capacity to recognize members of familiar categories [18–20], but moreover, and consistent with previous findings [29, 30], may blur distinctions among members of unfamiliar categories, making it easier to lump them as being of the same kind.

## Experiment 2: Subordinate categorization of familiar and unfamiliar race targets

The goal of Experiment 2 was to test a further implication of the perceptual narrowing of unfamiliar-race faces. Namely, if one is more likely to perceive unfamiliar than familiar exemplars as indistinguishable individuals, lumping the former together into a broad category, then one should be less likely to form subordinate categories within unfamiliar than familiar categories. Research on adults indicates that this is indeed the case [41–43], and there is some indirect evidence that this is true of infants as well. For instance, infants are better capable of discriminating between emotional expressions in own-race than in other-race faces [44], and infants look longer at an individual adult face over an infant face [45], and at a female over a males’ face [39, 40], but only if the faces are from their own-race. In other words, just as 3-month-olds infants show a preference for looking at an individual from their own race over an individual from another race [22, 32, 33], so they also show preferential looking at individuals of different “types” when they are from the infants’ race, but not when they are not. As discussed earlier, these findings nevertheless, do not ascertain that indeed infants perceived the individual faces as representatives of different types [24]. For instance, they do not establish that infants represented the adult face and the infant face as exemplars of two distinct sub-categories of people: “adults” and “infants”. To the best of our knowledge, only one study assessed infants’ capacity to form subordinate social categories, and found that by 12-months, infants could form gender categories of familiar-race faces [27]. The goal of Experiment 2 was to assess this hypothesis directly, by *comparing* infants’ capacity to form subordinate categories of familiar- vs. unfamiliar-race faces.

Another important feature of Experiment 2, was that differently from Quinn et al. [27], instead of assessing the formation of “conventional” categories (e.g., gender), we targeted novel arbitrary categories, i.e., ones based on shirt-color. In Experiment 2, infants were familiarized to White (or Black) adults wearing red shirts, and their categorization capacity was assessed by measuring their looking time at two other White (or Black) adults, one wearing a red and the other a blue shirt. One important theoretical motivation for employing arbitrary categories in this design is that they are socially meaningless. In other words, it should make no difference to an infant if a person wears blue or red. In contrast, it should–and does–make a difference if a person is male or female. Thus, whereas socially meaningful and labelled categories–such as gender–might be readily recognized by infants, arbitrary categories based on shirt color should not. For instance, “White men” and “White women” might exist *as distinct categories* from fairly early on; “White men wearing red” and “White men wearing blue” might not. Consequently, whereas exposure to familiar, socially meaningful, and labelled categories–such as gender–might explain the advantage of subordinate categorization of own-race vs. other-race faces, these factors do not apply to arbitrary categories. And thus, if we were to find a difference in subordinate categorization capacity along such a dimension, it would have to be due to factors other than the relative familiarity of the socially meaningless subordinate categories assessed here.

Given that the two test pictures (e.g., a White adult wearing a red-shirt and a White adult wearing a blue-shirt) did not differ in their familiarity to infants, we had no substantive reason to suspect that infants would have an a priori preference for one over the other–differently from the case in Experiment 1. Thus, we followed standard practices in the categorization literature [see for instance, 34] and did not include a baseline condition in Experiment 2. In addition, given the null findings in Experiment 1 regarding targets’ gender and labeling, in Experiment 2 all exemplars were men, and all infants were tested in a label condition. The hypothesis was that if subordinate categorization is harder when targets are from an unfamiliar than a familiar category, then infants would be less likely to discriminate between red and blue shirt wearing Black adults, than between red and blue shirt wearing White adults.

### Method

#### Participants

Thirty-four Israeli White 11-month-old infants (19 girls; mean age = 10.7 months; range = 9.2 months to 12.8 months) constituted the final sample of participants. None had participated in the previous experiment. Nine additional infants were excluded due to fussiness (6) or experimenters’ error (3). Criteria for inclusion, recruitment process, and testing conditions, were all identical to those adopted in Experiment 1. As in Experiment 1, sample size was planned in advance, determined according to previous studies in this area [28, 30, 36]. These studies reported effect sizes of approximately *Cohen’s f* = 0.4, and thus using this as an estimate, and setting α = 0.05, our sample sizes rendered an estimated power of approximately 0.62.

#### Design

Infants were assigned to one of two categorization conditions: White (*n* = 18) or Black (*n* = 16) targets.

### Materials and procedure

The main difference between Experiments 2 and 1 had to do with the stimuli presented. In Experiment 2, infants watched a sequence of White (or Black) men all wearing red shirts, followed by a test trial presenting two new White (or Black) men, one wearing a red shirt and the other a blue shirt (see Fig 1’s note for a detailed description). All other methodological details were identical to Experiment 1. Given that the test trial involved exemplars from the same race, we adopted the standard dependent measure of categorization for cases where there is no hypothesis for a particular a priori preference. Namely, we used the percentage of looking time at the contrasting category test exemplar (out of the total looking time at both test exemplars), with a value higher than 50% indicating categorization [see for instance, 35, 30]. Although this analysis is somewhat different than the one conducted in Experiment 1, it is in fact, the conventional measure used in the literature for examining infants’ categorization capacity [see for example: 27–30, 35–37]. Hence, although the studies were not preregistered, this analysis was planned beforehand, and researcher degrees of freedom were not abused.

### Results and discussion

**Fig 3:**
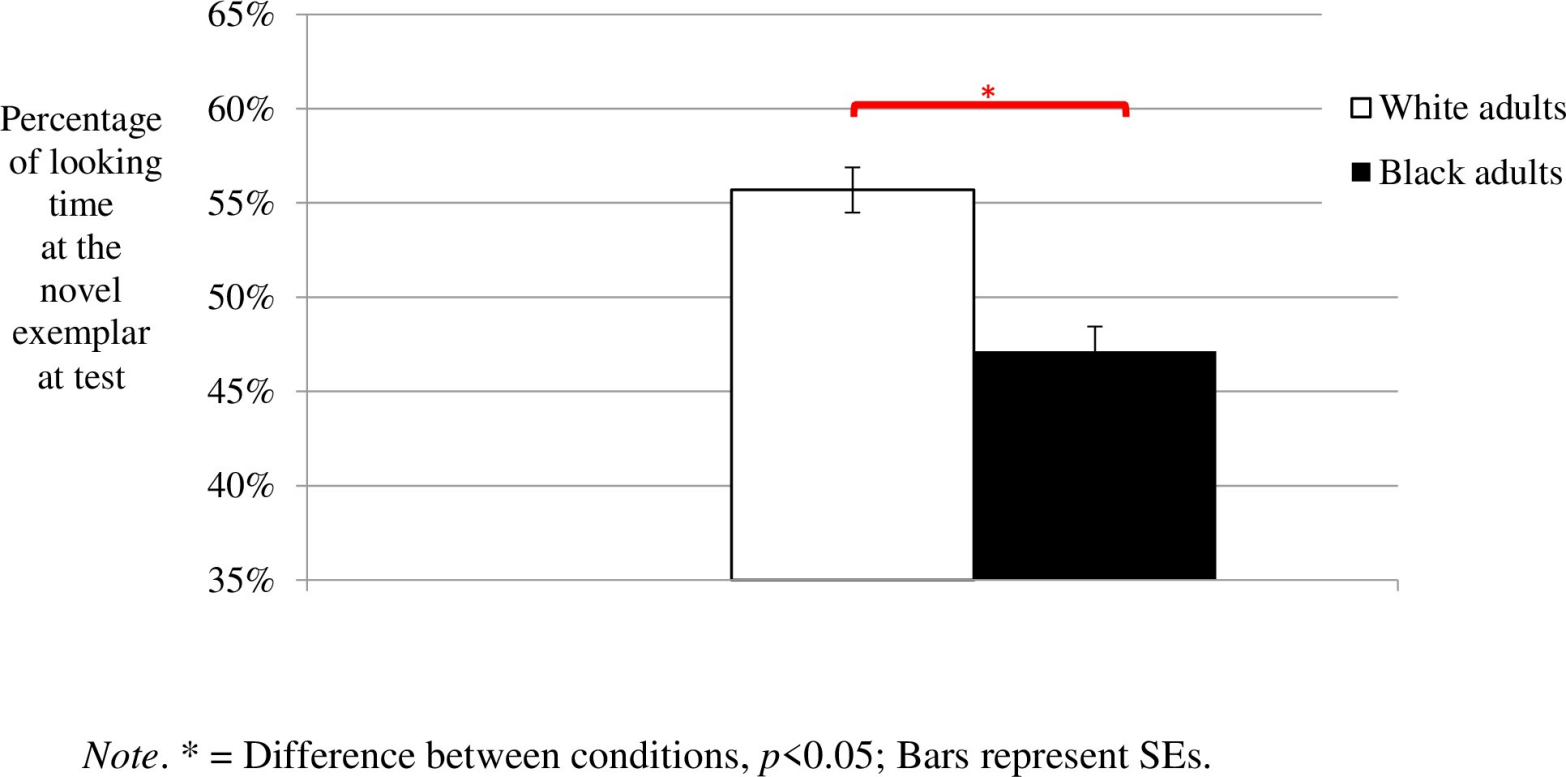
Results of Experiment 2 (Shirt-color).

An independent samples t-test comparing between the two conditions (Black vs. White targets), using the percentage of looking time at the contrasting category exemplar as the dependent measure, revealed a significant effect, *t*(32) = 2.334, *p* = 0.026, *Cohen’s d* = 0.825, *1-β* = 0.759. As can be seen in Fig 3, infants looked significantly longer at the contrasting category exemplar in the White targets condition than in the Black targets condition. In fact, one sample t-tests against a 50% criterion revealed that whereas infants in the White targets condition looked at the novel test exemplar longer than expected by chance (*M* = 56%, *SE* = 2.5%; *t*(17) = 2.267, *p* = 0.037, *r* = 0.481), infants in the Black targets condition looked at the novel exemplar as expected by chance (*M* = 47%, *SE* = 2.7%; *t*(15) = -1.072, *p* = 0.301, *r* = 0.266).

As a final analysis, we classified infants as to whether they looked at the contrasting test exemplar more (“categorizers”) or less (“non-categorizers”) than 50%. Non-parametric tests comparing the number of categorizers between conditions revealed a significant difference, *χ*^*2*^(1, *n* = 34) = 4.153, *p* = 0.042. In fact, whereas the proportion of categorizers in the White targets condition was significantly different from chance distribution (78%; *χ*^*2*^(1, *n* = 18) = 5.556, *p* = 0.018), this was not the case in the Black targets condition (44%; *χ*^*2*^(1, *n* = 16) = 0.250, *p* = 0.617). In sum, infants only succeeded in forming subordinate categories based on shirt-color when that cue was applied to familiar exemplars.

## General discussion

The goal of the present experiments was to assess the early emergence of a process arguably underlying inter-group biases in adults, namely, the tendency to view out-group members, or members of unfamiliar groups, as indistinguishable exemplars of a category. Given the mounting evidence on infants’ social categorization capacities and inter-group biases, we asked whether such a tendency would be present in infants as well. The experiments showed that 11-month-old infants’ capacity to represent social categories is indeed influenced by the familiarity of the to-be-categorized exemplars.

In Experiment 1, we found an asymmetry in the way White infants represented Black adults–their unfamiliar racial group–compared to White adults. Although this asymmetry in infants’ tendency to represent categories based on Black vs. White faces replicates previous findings on other social or racial categories [27–29], the present experiment is the first to show that the asymmetry is indeed due to the process of categorization, above and beyond any a priori preferences infants might have regarding exemplars of the categories. In particular, similar to previous studies assessing infants’ looking preferences at exemplars of familiar vs. unfamiliar categories, we too found that by 11-months of age, infants dedicate more time looking at exemplars from unfamiliar than familiar categories [22, 33]. Nevertheless, when exposed to a series of exemplars from an unfamiliar category, but not when exposed to exemplars from a familiar category, this preference disappeared.

The comparison between familiarization conditions and a baseline condition was crucial for more conclusively inferring that a process of categorization took place. To use an example from another domain, if infants were to look longer at a carrot than at an apple, we would not be able to conclude that they have the categories “fruit” and “vegetables”. But if after seeing beets, celery, lettuce, cucumber, and so on, their relative looking time at a carrot–when paired with an apple–significantly dropped, then we would feel more justified to infer that they indeed had the category vegetable, as exclusive of apples. This is the rationale underlying most studies assessing categorization as opposed to sheer discrimination.

In fact, if all we had was the difference between the two familiarization conditions, we could have reached the exact opposite conclusion from the one drawn here. Namely, we would have observed that when exposed to a series of White exemplars, infants looked proportionately longer at the contrasting category exemplar (in that case, the Black exemplar), than when exposed to a series of Black exemplars (thus looking at the White exemplar). In other words, we could have concluded that categorization more readily followed exposure to familiar exemplars than exposure to unfamiliar exemplars. As we have argued, following others in the literature [27, 28, 34], that pattern, however, could have simply reflected 11-month-olds’ a priori preferences to look longer at a Black face than at a White face. And indeed, the 11-month-olds tested in our baseline condition manifested precisely this a priori preference. It is only by comparing the patterns found in each familiarization condition to the baseline condition that we could conclude that only familiarization to Black exemplars significantly altered infants’ a priori preference.

In a sense, the finding that the preference did not change after familiarization to familiar exemplars is not surprising. After all, what are nine more White adults for a White infant raised in a predominantly White environment? Why should that change their prior expectations about what people look like and how people should be grouped? In fact, these prior expectations could reflect two alternative possibilities. One possibility is that by 11-months of age, White infants indeed have a category of “White people” [see for instance, 31], and thus upon encountering multiple exemplars of that category, they can readily process their individuality. A second possibility is that for White infants raised in a predominantly White environment, “whiteness” is not a feature by which people are differentiated and grouped. Even White adults seem “blind” to the race of White people, picking up race as a feature more readily when exposed to Black than to White exemplars [46]. In this interpretation, our infant participants exposed to White adults during familiarization were simply seeing “people”, and only when exposed at test to a person of a different race than they are used to seeing, their attention was caught by it.

Interestingly, however, neither of the above possibilities capture how Black adults were perceived. Exposure to nine Black adults sufficed to alter the a priori salience of Blacks *and Whites* in White infants’ eyes. Such an exposure was sufficient to substantially increase infants’ proportionate looking at a White adult, making it as interesting as a Black adult. The argument is that the exposure to nine Black exemplars drove infants to recognize the similarity among them, and their dissimilarity from White people. Familiarization to Black exemplars made race a discriminant feature. In this regard, we believe that the process underlying this realization is akin to a process of habituation. Namely, as our White infant participants got further exposure to relatively novel *and different* stimuli (faces of Black adults), they started to notice some similarity among the stimuli, and eventually got habituated to whatever feature of similarity they picked up. The infants saw the faces we as adults call “Black”, as being sufficiently similar to each other so that the tenth one they saw (at test), was indeed considered “more of the same”, leading to a significant drop (compared to baseline) in infants’ interest in it. One important caveat to this conclusion is that the lack of difference between the two familiarization conditions, suggests that the identical exposure to 9 faces that infants in these two conditions underwent (albeit different faces), might have similarly affected infants’ looking tendencies. Thus. the decrease–relative to baseline–in infants’ looking at the test Black face after being familiarized to Black faces, may be in part explained by a general decrease in interest in faces. Nevertheless, it was only in this condition, and not in the familiarized-to-White condition, that such a decrease was statistically reliable.

A further interesting datum from the present work that may be relevant to the above points, had to do with the observed variance in infants’ looking time between the two familiarization conditions of Experiment 1. In particular, whereas the range of proportionate looking time at the Black test exemplar for infants in the White familiarization condition was somewhat narrow (43%-68%), the range among infants in the Black familiarization condition was quite wide (24%-78%). Resonating with the above discussion, exposure to a series of Black faces was likely much more “disruptive” and novel to our White participants, than exposure to a series of White faces, leading to greater individual differences in the strategies for processing such stimuli.

Taken together, as hypothesized, the asymmetry in categorization is consistent with the findings on the development of the other-race effect [18–20]. By 9-months of age, faces from familiar races are more readily recognized than from unfamiliar ones, arguably because infants more competently pick up subtle individuating features that distinguish among the former. Complementarily, this enhances a propensity to view faces of unfamiliar social categories as more alike. One interesting implication of this correspondence is that attempts at reducing the development of the other-race effect in infants by exposing them to other-race faces [47–49], might also weaken the asymmetry in the representation of categories. This is especially valuable given the potential of such interventions to attenuate young children’s racial bias [16].

The second major finding of the present studies is that infants in Experiment 2 found it easier to represent subordinate categories of a familiar race than of an unfamiliar one. Previous studies had shown that infants’ preferences and sensitivity for faces of different genders, ages, or emotional expressions is particularly prevalent when the faces are from infants’ familiar race [39, 40, 44, 45]. Experiment 2 showed that familiar-race faces are also more readily broken down into subcategories. Note that this is the case irrespective of whether infants represented the White exemplars as being simply familiar, as belonging to the category “Whites”, or belonging to the category “people”. In all these cases, it would have been easier for infants to detect the unique features of the red-wearing White adults, than of the novel and to-be-categorized red-wearing Black adults. Figuring out how exactly infants construed the White exemplars is important, nevertheless, for understanding whether infants represented these subcategories as subordinate. For instance, it is possible that infants at this age have difficulty construing an embedded hierarchy of categories, with categories at multiple levels. Alternatively, it could be that highly familiar social categories indeed allow for the formation of subcategories. This is a valuable question for future research.

Further important points to draw from Experiment 2 have to do with the nature of the subordinate categories assessed. Instead of using “conventional” categories that infants might have encountered in their daily lives and have a unique social meaning (e.g., race or gender), here infants were asked to represent novel and arbitrary categories, based on shirt-color. One implication of this design feature is that infants’ higher capacity to represent the category “red-shirted White men” compared to “red-shirted Black men” evidently did not derive from the fact that the former was a prepotent category already accessible to infants, whether the latter was not. Rather, the differential competence had to derive from how infants processed individuals from familiar vs. unfamiliar races. In this regard, an additional implication of the present design is that the different processes undergone by infants when analysing familiar vs. unfamiliar exemplars was not reduced to the analyses of faces [cf.23]. Rather, the recognition of a face as familiar or unfamiliar arguably gave rise to further biases in the processing of the entire exemplars, in the former case allowing infants to pick out differences in shirt-color and in the latter not. In other words, even though infants could have simply noticed that there are two different colors of shirts being displayed at test, it mattered *who* was wearing the shirts.

An interesting goal for future studies is to explore the nature of these differential processes [see 11, for a discussion]. One possibility is that the processing of familiar faces being more efficient, it is less resource intensive, and thus frees attentional and memory capacities for more fine-grained and peripheral analyses. A second intriguing possibility is that motivational factors are involved, with even infants becoming more engaged in identifying discriminating features of in-group vs. out-group faces [see 50, for a discussion]. The latter possibility is also relevant for relating the phenomenon uncovered here, with the more general and abstract phenomenon of the out-group homogeneity effect. Namely, to the extent that infants are less engaged in getting to know out-group members, the asymmetry revealed here regarding sensitivity to physical features may extend to sensitivity to psychological features as well. This may also carry an important practical implication. Specifically, whereas previous studies with adults have concluded that the sheer capacity to form social categories is not necessarily related to intergroup attitudinal biases [8, 9], studies with children revealed that the capacity for multiple classification, i.e., seeing individuals as belonging to various categories, is related to positive attitudes [51, 52]. Reinforcing an understanding that individuals belong to multiple social categories may thus function as an important intervention tool.

The present experiments also generated two somewhat surprising findings. First, we found that labeling did not have a significant effect on racial categorization. As noted in the Introduction, several studies had found that the provision of labels during familiarization facilitated infants’ formation of object and animal categories [see 53, for a review]. One possible explanation for the present null-finding is that whereas in the domains of objects and animals, the primary latent categories that labels presumably highlight are narrowly instantiated in the familiarization exemplars (e.g., “rabbits”, “pigs”, “birds”), in the social domain, the primary latent category might be broader, i.e., “people” [see 54, for support for this notion]. Consequently, and as we argued earlier, labeling White adults as done here, would not have highlighted the fact that they are all *White* or *Black*, but rather that they are all *people*, which would then not facilitate discriminating between White and Black people. Nevertheless, with development, and increasing exposure to and understanding of specific social category labels (e.g., “White” and “Black”), labels can facilitate categorization of various social groups [cf. 38]. As Waxman has suggested, labels may function as invitations to form categories, encouraging infants and toddlers to search for features shared by referents of a common label. It is possible, nonetheless, that for labels to have such an effect across a wide range of domains, and across various levels of categorical abstraction (i.e., subordinate, basic, and superordinate), children have to achieve more sophisticated linguistic and conceptual capacities. Undoubtedly, these capacities develop substantially in the second year of life [e.g., 55, 56].

The second somewhat surprising finding was the lack of significant effects involving targets’ gender, on infants’ capacity to represent racial categories. One possible explanation for this null-finding is that by 11-months of age, the facilitatory effect of female faces to infants’ recognition capacities [39, 40] starts to fade away. It is also important to point out in regard to both of these null-findings, that although we believe the design of the present studies had sufficient power to detect the main effects of these two variables (labeling and targets’ gender), it may not have had sufficient power to detect possible two- or three-way interactions among the manipulated factors. It could be an interesting question for future studies to pursue such interactions, for instance, whether labeling may have an effect in the racial categorization of presumably easier to recognize faces (women) but not of harder ones (men), or vice-versa.

A broader point about the present studies has to do with their generalizability, both in terms of racial groups, and with respect to other kinds of social categories. On the first point, note that one limitation of the present research is that it only included infants for whom White people are familiar and Black people are unfamiliar. Hence, the effects that are attributed here to the racial familiarity of the faces could be due to other differences between White and Black faces. Thus, an open question for future research is whether the effects reported here would be exhibited by infants who more regularly encounter Black faces in their natural environments.

As for the second point regarding generalizability, the present studies operationalized familiarity via racial groups. Arguably, however, the phenomena revealed here might apply to other kinds of social groups. As recently proposed by Lee, Quinn, & Pascalis [57], the underlying developmental process of category representation might indeed be more general in nature, and not specific to representing racial categories and or picking out race as a category defining feature (see for instance, Pietraszewski, Cosmides, & Tooby, [58], for a general argument along these lines). Consequently, we should find evidence for such a process even in the representation of categories based on arbitrary features–something future studies can pursue.

In conclusion, the present studies reveal that infants view unfamiliar people as homogeneous, thus: a) facilitating the representation of them as a group, and b) hindering the recognition of their sub-groups.

## Supporting information

S1 Data (XLS)

S2 Data (XLS)
